# Status of benzimidazole resistance in intestinal nematode populations of livestock in Brazil: a systematic review

**DOI:** 10.1186/s12917-017-1282-2

**Published:** 2017-11-25

**Authors:** Lauren Hubert Jaeger, Filipe Anibal Carvalho-Costa

**Affiliations:** 1 Laboratório de Epidemiologia e Sistemática Molecular, Instituto Oswaldo Cruz, Fundação Oswaldo Cruz, Pavilhão Leônidas Deane, sala 308, Avenida Brasil 4365, Manguinhos, Rio de Janeiro, RJ 21040-900 Brazil; 2 Fundação Oswaldo Cruz, Rua Magalhães Filho, 519, Teresina, Piauí 64000-128 Brazil

**Keywords:** Benzimidazole, Anthelmintic resistance, Nematodes, Livestock, Brazil

## Abstract

**Background:**

Benzimidazoles (BZ) are a class of drugs widely used in veterinary and human medicine, creating a great selection pressure and the emergence of BZ resistance. We conducted a systematic review to assess the status of resistance and/or effectiveness reduction of BZ drugs in animal nematodes in Brazil, and make information accessible to the scientific community, as many studies are published in Portuguese. PubMed, SciELO Brasil, LILACS/Bireme, GNTD database, and Google Scholar were searched with no language restrictions.

**Results:**

A total of 40 studies met our eligibility criteria (from the year 1989 forward). Sheep was the host most frequently analysed, and albendazole was the most frequently drug studied. The majority of studies (75.7%) showed that BZ drugs are insufficiently active (FECRT <80%) against nematode parasites of livestock. The mean FECRT for fenbendazole, thiabendazole, albendazole, mebendazole, oxfendazole, and ricobendazole were 71.8%, 71.8%, 58.6%, 53.9%, 46.9%, and 41.5%, respectively. It was observed through linear regression that FECRT is significantly reduced over time between 2007 and 2014 (*R* = −0.653 *p* = 0.021) for the treatment of cattle with BZ, suggesting progressive loss of effectiveness and increased resistance for these hosts.

**Conclusions:**

The scenario of BZ resistance in nematode populations in Brazil is not favourable. Given the high cost of drug discovery and development, it is urgent to implement control measures and to monitor the effectiveness/resistance to nematodes in livestock in Brazil.

## Background

Livestock production is undermined by intestinal parasitic diseases [1]. The high prevalence of parasitic infections and the difficulty of carrying out effective control of these parasites in livestock can cause huge economic losses in production [2]. In addition to the damage caused by high mortality rates, intestinal parasites impact growth performance, reduce milk production and lead to low fertility [3]. The most important genera affecting livestock in Brazil include *Haemonchus, Trichostrongylus, Oesophagostomum, and Cooperia* [3]. The cost of veterinary products is approximately 15 billion US dollars annually worldwide, and 27% of this cost is represented by parasiticides. In Brazil, parasiticide purchases constitute 42% of the total volume of veterinary sales, representing 700 million US dollars annually [3]. Brazilian cattle herds reached 211,764 million animals in 2013, comprising the world’s largest commercial herd. Brazilian sheep and goat herds comprised 17,291 million and eight million animals, respectively [4].

Benzimidazoles (BZ) are a class of drugs with activities against fungi, protozoa, and helminths [5, 6] and are widely used in veterinary and human medicine. The introduction of thiabendazole (THI) in the 1960s - the prototype of the first generation of benzimidazoles - provided a breakthrough in the treatment of diseases, allowing for the development of several other members of this class [5]. The primary mode of action of these drugs involves their interaction with the cytoskeletal protein β-tubulin, which together with α-tubulin constitutes the main component of microtubules [7]. The BZ drugs have many benefits, including the following: i) selectivity and relatively low mammalian toxicity; ii) broad spectrum of activity; iii) high efficacy; iv) ease of administration; and v) low cost [5, 7–9]. For this reason, BZ drugs are widely used in livestock and are currently being employed in human MDA strategies. The success of anthelmintic treatment in the management and control of parasitic infections in livestock in the years following the development of BZ led to frequent and indiscriminate use of these drugs, thereby creating a great selection pressure in multiple species of nematodes [10]. This has the potential to select for parasite genotypes that are resistant to anthelmintics [11]. Drug resistance in any organism is defined by a change in the drug’s pharmacokinetics and pharmacodynamics (absorption, distribution, metabolism, excretion, and site of action) [5] that allows some individuals in a population to tolerate doses of a given compound that would not normally be tolerated.

In this systematic review, we assess the status of resistance and/or effectiveness reduction of benzimidazole drugs in livestock nematodes in Brazil to review the history of BZ resistance in the country, generate data to enable monitoring and verification of the spread of BZ resistance, and make information accessible to the scientific community, as many studies are published in Portuguese.

## Methods

### Data sources and inclusion/exclusion criteria

Surveys assessing BZ resistance pertaining to animal intestinal nematodes in Brazil were extracted from five electronic databases: PubMed/NCBI (US National Library of Medicine National Institutes of Health/National Center for Biotechnology Information Search database), SciELO Brasil (Scientific Electronic Library Online), LILACS-Bireme (*Biblioteca Virtual em Saúde* – BIREME/PAHO/WHO), GNTD database (Global Neglected Tropical Diseases database), and Google Scholar. The search was performed on November 21st, 22nd, and 28th, 2015, using the terms: “resistance”, AND “benzimidazole”, OR “albendazole”, OR “mebendazole”, AND “Brazil”. No language restrictions were made. Duplicate papers were removed. The PRISMA guideline/checklist was used to construct the systematic review [12].

Studies were eligible for inclusion if they met the following criteria: i) evaluated the BZ resistance/efficacy in nematode parasites in livestock hosts; ii) studied natural infections; iii) showed BZ resistance/efficacy of at least one BZ anthelmintic; iv) used at least one technique to detect BZ resistance/effectiveness; and v) were published in scientific journals with an International Standard Serial Number (ISSN). Congress abstracts, theses, and dissertations were not included. The exclusion criteria were as follows: i) articles that explored the BZ resistance only associated with other drug classes (e.g., BZ + macrocyclic lactones and other associations); ii) studies evaluating BZ resistance on fungi or other microorganisms; and iii) works demonstrating only experimental infections or in vitro tests.

“Grey literature” was accessed to enrich the text but was not included in the systematic review.

### Data extraction, analysis, and quality assessment

Once selected, the following data were extracted from each paper and entered into a Microsoft Office Excel database: author names, journal, publication year, language, state and city in which the study was performed, host types, number of hosts, BZ drugs, parasitological techniques, counts of eggs per gram (epg) of faeces, parasites genus/species found, control group, if animal was dewormed and for how long, BZ resistance-related single nucleotide polymorphisms (SNPs) found, efficacy, cure rate, and reinfection rate. The studies were categorized into five quality levels (1 to 5; data not shown) based on the detail of the herds, number of animals evaluated, drugs evaluated, number and quality of the parasitological techniques used, and FECRT calculation.

### Faecal egg count reduction test analysis

An assessment of treatment efficacy was performed by analysing the **F**aecal **E**gg **C**ount **R**eduction **T**est (FECRT syn. Egg reduction rate/ERR) results. When the studies did not present FECRT results, the values were calculated based on the eggs per gram of faeces before and after treatment, according to [13]. For the interpretation of the FECRT results in livestock, the following criteria were used: FECRT > 98%, highly effective; FECRT 90–98%, effective; FECRT 80–89%, moderately effective; and FECRT < 80%, insufficiently active [14].

The SPSS® Statistic Software v.20 (IBM Corp., Armonk, USA) was used to simple linear regression analysis, with a statistical significance of 5% (*p* = 0.05). We employed the general software Diva-GIS v.7.5.0.0 for map construction (downloaded free from the website: http://www.diva-gis.org).

## Results

The search resulted in the gathering of 9176 files (articles or other texts). After applying the inclusion and exclusion criteria, 40 scientific articles were selected (Table 1). Most of the studies were published in Portuguese (23/40, 57.5%), and 17 (42.5%) studies were published in English. This review includes articles conducted from the year 1989 forward.

**Table 1:** List of studies assessing benzimidazole resistance in livestock hosts in Brazil, from 1989 to 2015

Author ^a^ year [reference number] Journal	State	BZ drug	Host	Diagnostic Approach			Nematode genus
				Parasitological technique		Molecular technique (SNP detection)	
				McMaster epg	Culture		
Ahid et al. 2007 [43]	AL	ALB	Goat	Y	Y	N	*Haemonchus, Strongyloides*
Acta Veterinaria Brasilica							
Amarante et al. 1992 [44]	SP	OXF	Sheep	Y	Y	N	*Haemonchus, Trichonstrongylus*
Brazilian Journal of Veterinary							
Araujo et al. 2008 [45]	RN	RIC	Equine	Y	N	N	NA
Acta Veterinaria Brasilica							
Borges et al. 2010 [46]	PR	OXF	Equine	Y	Y	N	Cyathostominae
Ciência Animal Brasileira							
Borges et al. 2015 [15]	BA	ALB	Goat	NI	Y	N	*Haemonchus, Trichonstrongylus*
Pesquisa Veterinária Brasileira							
Brasil et al. 2012 [47]	MG	ALB	Cattle	Y	Y	Y	*Haemonchus*
International Journal for Parasitology	SP		Goat				
	SC		Sheep				
Bruhn et al. 2012 [48]	MG	ALB	Cattle	Y	N	N	NA
Acta Tecnológica							
Cezar et al. 2010 [16]	RS	ALB	Sheep	Y	Y	N	*Haemonchus, Trichonstrongylus, Ostertagia*
Veterinary Parasitology							
Coelho et al. 2010 [17]	RN	ALB	Goat	Y	Y	N	*Haemonchus, Trichonstrongylus*
Ciência Animal Brasileira							
Cunha Filho et al. 1998 [49]	PR	ALB	Sheep	Y	Y	N	*Haemonchus, Strongyloides, Trichonstrongylus, Ostertagia, Oesophagostomum, Cooperia, Bunostomum*
Semina Ciências Agrárias							
da Cruz et al. 2010 [18]	RJ	ALB	Sheep	Y	N	N	NA
Veterinary Parasitology							
das Neves et al. 2014 [30]	SP	ALB	Cattle	Y	Y	N	*Haemonchus, Trichonstrongylus, Oesophagostomum, Cooperia*
Veterinary Parasitology							
de Souza et al. 2012 [50]	MG	ALB	Ostrich	Y	Y	N	*Libyostrongylus*
Veterinary Parasitology							
dos Santos et al. 2014 [51]	CE	OXF	Sheep	Y	Y	Y	*Haemonchus, Trichonstrongylus, Oesophagostomum*
Veterinary Parasitology							
Duarte et al. 2012 [52]	MG	ALB	Sheep	Y	Y	N	*Haemonchus, Strongyloides, Trichonstrongylus, Oesophagostomum, Cooperia*
Pesquisa Veterinária Brasileira							
Echevarria et al. 1996 [53]	RS	ALB	Sheep	NI	Y	N	*Haemonchus, Trichonstrongylus, Ostertagia*
Veterinary Parasitology							
Farias et al. 1997 [54]	SP	ALB	Sheep	Y	Y	N	*Haemonchus, Trichonstrongylus, Ostertagia*
Veterinary Parasitology		MEB					
		OXF					
Hammerschmidt et al. 2012 [20]	SC	ALB	Goat	Y	Y	N	*Haemonchus, Trichonstrongylus, Oesophagostomum*
Brazilian Journal of Veterinary Research and Animal Science							
Klauck et al. 2014 [55]	SC	ALB	Sheep	Y	Y	N	*Haemonchus, Trichostrongylus, Cooperia, Teladorsagia*
Annals of the Brazilian Academy of Sciences							
Lima et al. 2010 [56]	PE	ALB	Goat	Y	Y	N	*Haemonchus, Trichonstrongylus, Oesophagostomum*
Ciência Animal Brasileira			Sheep				
Lima et al. 2010 [57]	PE	ALB	Goat	Y	Y	N	*Haemonchus, Strongyloides, Trichonstrongylus, Oesophagostomum*
Pesquisa Veterinária Brasileira							
Melo et al. 1998 [58]	CE	OXF	Sheep	Y	Y	N	*Haemonchus, Trichonstrongylus, Cooperia, Trichuris*
Ciência Animal							
Melo et al. 2003 [59]	CE	OXF	Goat	Y	Y	N	*Haemonchus, Trichonstrongylus, Oesophagostomum*
Ciência Rural			Sheep				
Niciura et al. 2012 [60]	SP	ALB	Sheep	N	N	Y	*Haemonchus*
Veterinary Parasitology							
Nunes et al. 2013 [61]	MG	ALB	Cattle	Y	N	Y	*Haemonchus*
Revista Brasileira de Parasitologia Veterinaria	SP		Goat				
			Sheep				
Pereira et al. 2008 [62]	RN	ALB	Goat	NI	Y	N	*Haemonchus, Strongyloides, Trichonstrongylus, Oesophagostomum*
Acta Veterinaria Brasilica			Sheep				
Ramos et al. 2002 [63]	SC	ALB	Sheep	Y	Y	N	*Haemonchus, Trichonstrongylus, Ostertagia*
Ciência Rural							
Rodrigues et al. 2007 [64]	PB	ALB	Goat	Y	Y	N	*Haemonchus*
Pesquisa Veterinaria Brasileira							
Santos et al. 2014 [65]	RS	OXF	Cattle	Y	Y	N	*Haemonchus, Trichonstrongylus, Ostertagia, Cooperia, Bunostomum*
Revista Portuguesa de Ciências Veterinarias							
Sczesny-Moraoes et al. 2010 [66]	MS	ALB	Sheep	Y	Y	N	*Haemonchus, Strongyloides, Trichonstrongylus, Cooperia*
Pesquisa Veterinária Brasileira							
Soutelo et al. 2007 [67]	SP	ALB	Cattle	NI	Y	N	*Haemonchus, Trichonstrongylus, Oesophagostomum, Cooperia*
Veterinary Parasitology							
Soutelo et al. 2010 [68]	SP	ALB	Cattle	Y	Y	N	*Haemonchus, Oesophagostomum, Cooperia*
Revista Brasileira de Parasitologia Veterinária							
Souza et al. 2008 [69]	SC	ALB	Cattle	Y	Y	N	*Cooperia*
Ciência Rural							
Souza et al. 2013 [70]	PB	ALB	Goat	Y	N	N	NA
Agropecuária Científica do Semiárido							
Thomas-Soccol et al. 1996 [21]	PR	ALB	Sheep	Y	N	N	NA
Veterinary Record							
Thomas-Soccol et al. 2004 [22]	PR	OXF	Sheep	NI	Y	N	*Haemonchus, Trichonstrongylus, Oesophagostomum, Ostertagia, Cooperia*
Brazilian Archives of Biology and Technology							
Veríssimo et al. 2012 [71]	SP	ALB	Sheep	NI	Y	N	*Haemonchus, Strongyloides, Trichonstrongylus, Oesophagostomum, Cooperia*
Veterinary Parasitology							
Vieira and Cavalcante 1999 [72]	CE	OXF	Goat	Y	Y	N	*Haemonchus, Oesophagostomum*
Pesquisa Veterinária Brasileira							
Vieira et al. 1989 [73]	CE	ALB	Goat	NI	Y	N	*Haemonchus, Trichonstrongylus, Oesophagostomum*
Pesquisa Agropecuária Brasileira		FEN					
		OXF					
		THI					
Vieira et al. 1989 [74]	CE	ALB	Goat	NI	Y	N	*Haemonchus, Strongyloides*
Boletim de Pesquisa Embrapa		FEN	Sheep				
		OXF					
		THI					
Total = 40							

^a^listed alphabetically. *ALB* albendazole, *FEN* fenbendazole, *MEB* mebendazole, *OXF* oxfendazole, *RIC* ricobendazole, *THI* thiabendazole. *Epg* eggs per gram of faeces, *AL* Alagoas, *BA* Bahia, *CE* Ceará, *MG* Minas Gerais, *MS* Mato Grosso do Sul, *PB* Paraíba, *PE* Pernambuco, *PR* Paraná, *RJ* Rio de Janeiro, *RN* Rio Grande do Norte, *RS* Rio Grande do Sul, *SC* Santa Catarina, *SP* São Paulo. *Y* Yes, *N* No, *NI* Not informed, *NA* Not applied

**Fig. 1:**
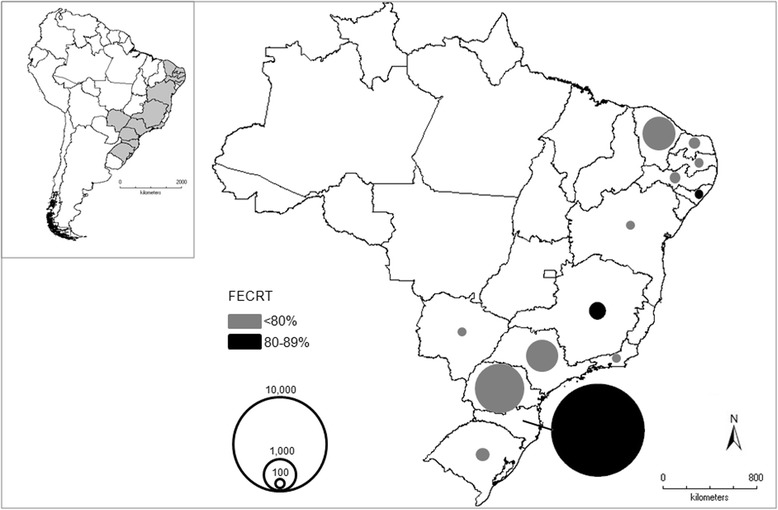
Mean FECRT in livestock of the Brazilian states. Area of the circle is proportional to number of animals

Data from 13 Brazilian states were analysed in the studies (Fig. 1). The largest number of studies was performed in Northeast (14/40, 32.6%), followed by Southeast (32.5%), South (30.0%), and Centre West (2.5%). The states most frequently analysed were the following: São Paulo (9/40, 22.5%), Ceará (15.0%), and Minas Gerais and Santa Catarina (12.5%) (Table 1).

In livestock, sheep were the host most frequently analysed (23/40, 57.5%) (Table 2), followed by goats (37.5%), cattle (17.5%), and others (equines and ostrich, 7.5% each). The BZ drugs tested in livestock were ALB (31/40, 72.1%), oxfendazole (OXF) (27.5%), fenbendazole (FEN) (4.7%), THI (4.7%), and MEB (2.3%), and ricobendazole (RIC) (2.3%).

**Table 2:** BZ effectiveness parameters in livestock nematodes in Brazil

Drug	Parameters	Host			Overall livestock^a^
		Cattle	Goat	Sheep	
BZ^b^	N studies (%)	7 (17.5)	15 (37.5)	23 (57.5)	40
	N hosts	3417	1697	11,342	16,531
	FECRT (%)				
	Minimum	7.3	20.8	0	0
	Maximum	95.9	90.0	90.0	100
	Mean	75.3	64.8	47.0	55.0
ALB	N studies (%)	7 (22.6)	11 (35.5)	16 (51.6)	31
	N hosts	3417	604	8878	12,915
	FECRT (%)				
	Minimum	7.3	29.5	0	0
	Maximum	95.7	90.0	90.0	100
	Mean	75.4	68.1	55.1	58.6
OXF	N studies (%)	0	4 (36.4)	7 (63.6)	11
	N hosts		1118	2464	3614
	FECRT (%)				
	Minimum		20.8	0	0
	Maximum		73.5	64.9	92.4
	Mean		56.6	29.7	46.9

^a^including cattle, goat, sheep, and others (equine and ostrich). ^b^including ALB, FEN, MEB, OXF, RIC, and THI

The majority of studies (75.7%) showed that BZ drugs are insufficiently active (FECRT <80%) against nematode parasites of livestock (Fig. 1). The mean FECRT for BZ drugs was 55.0% (Table 2); the mean FECRTs for FEN and THI was of 71.8%, the mean for ALB was 58.6%, the mean for MEB was 53.9%, the mean for OXF was 46.9%, and the mean for RIC was 41.5%. Five studies showed FECRTs lower than 1%. It is noteworthy that the most studied animal – sheep – showed the lowest mean FECRT for BZ drugs (mean FECRT = 47.0%) (Table 2). Only one study (2.5%) demonstrated that ALB is highly effective (FECRT > 98%) in ostrich against the nematode genus *Libyostrongylus*.

**Fig. 2:**
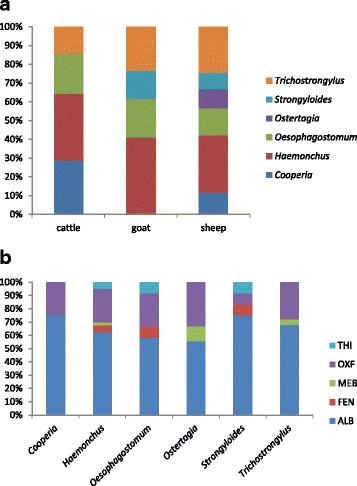
Number of studies that reported (**a**) the parasite species by host and (**b**) the most commonly used drug by parasite species, in Brazil, from 1989 to 2015

Among the techniques used to perform the eggs counts, the Gordon and Whitlock technique associated with the McMaster chamber was the most frequently used (75.0% of studies). The egg hatch test, as well as the FLOTAC technique, was used in only one study. Coproculture was performed in 33/40 (82.5%) of the studies to identify the nematode genus through morphological analyses of the larvae. Through coproculture, the following parasite genera were identified (Table 1): *Haemonchus* (32.2%), *Trichostrongylus* (21.7%), *Oesophagostomum* (16.1%), *Cooperia* (9.6%), *Strongyloides* (8.8%), *Ostertagia* (5.6%), and others *(Strongylus, Bunostomum, Teladorsagia, Trichuris, Libyostrongylus*, 5.6%). Sheep exhibited a greater diversity of parasites compared to other animal hosts (Fig. 2a), with the following genera identified: *Cooperia, Haemonchus, Oesophagostomum, Ostertagia, Strongyloides,* and *Trichostrongylus*. In Fig. 2b, it noted the number of studies that identified the parasite species and the studied drug. However, it was not possible to establish a relationship between a specific parasite genera and BZ effectiveness. Four studies (9.3%) used molecular techniques to evaluate potential BZ resistance. The characteristic mutation at codon F200Y of the beta-tubulin gene was the most frequently observed mutation (100%) in *Haemonchus* parasites, yet the F167Y mutation in the same gene was found in only two studies (50%).

**Fig. 3:**
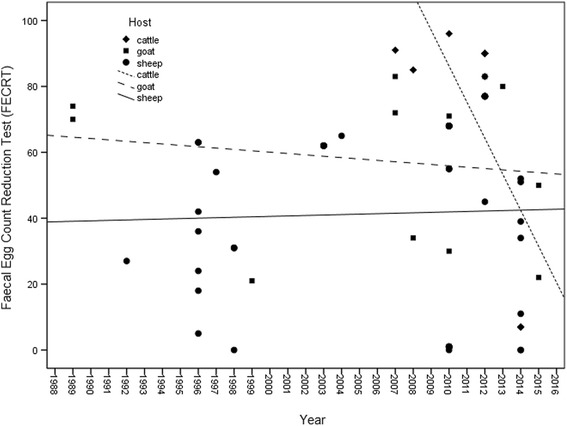
Simple linear regression analysis (lines) of Faecal Egg Count Reduction Test (FECRT) by time (years) reported in livestock in Brazil, from 1989 to 2015, stratified by host. Cattle *R* = −0.653 *p* = 0.021; goat *R* = −0.154 *p* = 0.633; and sheep *R* = 0.029 *p* = 0.820)

It was observed through linear regression that FECRT is significantly reduced over time between 2007 and 2014 (*R* = −0.653 *p* = 0.021) for the treatment of cattle with BZ, suggesting progressive loss of effectiveness and increased resistance for these hosts (Fig. 3).

## Discussion

This study presents a systematic review on a subject still under-explored in Brazil: BZ resistance in nematode parasites.

We observed that BZ resistance was widely disseminated in animal hosts in Brazil and demonstrated that BZ had lower effectiveness in sheep. BZ resistance in livestock has been widely distributed throughout the world since the development of the drugs in the early 1960s [7]. The extensive use of BZ led to an immense selection pressure on parasite populations, particularly in the gastrointestinal parasites of ruminants, most notably sheep [7]. Grazing animals defecate where they feed, and even after treatment, reinfection is common, leading to an overuse of BZs. In this context, anthelmintic drugs are often used as a single tool for nematode control, extensively and indiscriminately. Many farms provide various annual doses of BZ drugs to animals, allowing for a considerable selective pressure on parasitic nematodes, and the spread of resistance.

Many livestock studies report an inefficient management system, as well as, a lack of knowledge about the correct use and dosage of drugs and not respecting the interval time between dosage administrations [15–18]. This has a great impact on treatment efficacy. In Brazil, the SICOPA (***S*** *istema* ***I*** *ntegrado de* ***Co*** *ntrole* ***Pa*** *rasitário*) [19] consists of a set of strategies for the treatment of the flock to preserve the drug susceptibility characteristics and considers the epidemiological characteristics of the country [20]. However, the monitoring of drug efficacy is rarely used or even non-existent on some farms in Brazil [19]. Therefore, some measures must be implemented in farms in order to reduce the selective pressure and the spread of resistance to anthelmintics: i) establish the parasitological diagnosis; ii) determine the FECRT routinely, as well as the susceptibility of the host population (naïve, preparturition, post parturition); iii) weigh the animals to avoid underdosage; iv) anthelmintic drug rotation (annually); and v) anthelmintic treatments not administered at intervals shorter than 28 days [19–23].

Brazil is a major producer of animals and meat for exportation to the world market, approximately 230 million animals are produced annually [4]. Nonetheless, only 13 of the 27 Brazilian states were analysed for BZ resistance. Cattle production is well distributed in the country, especially in the states of the Midwest (33.6% of total production in the country) - specifically the states of Mato Grosso (13.4%) and Goiás (10.2%) - and the North Region is the second largest producer of cattle (21.2%) [4]. However, no work has been published reporting the effectiveness of BZ in these regions. Only one study was conducted in Mato Grosso do Sul (Midwest Region) and analysed sheep nematodes. The states of the Northeast and South Regions are the largest producers of sheep (56.5% and 30% of total production of the country, respectively), and the states of the Northeast are the main producers of goats, with 91.4% of the total production [4]. In these regions there was research available on BZ resistance of STHs in herds, both in sheep and goats. Nevertheless, the data shows us that there is a gap in knowledge - both in diagnosis and research - about the reality of resistance in livestock in the country.

The origin of BZ resistance in livestock has been speculated about. The animal migration and gene flow among nematodes [24], as well as spontaneous mutations [25] and the presence of rare alleles in the population [26], could be responsible for the spread of resistance among animal nematodes. Currently, there is concern about the possibility of the emergence of resistance to the drugs used in soil transmitted helminthes (STH) control; however, the large-scale mass drug administration strategy is generally the cornerstone of most STH control programmes [27, 28]. Until now, the degree of influence that resistance in livestock can have on the development and spread of resistance in human nematodes is unknown, particularly in nematodes with zoonotic potential, such as *Ascaris suum* and *Trichostrongylus*.

The egg count using the McMaster chamber, and several variations on the original technique, is the most frequently used technique to conduct the FECRT. The FECRT is an in vitro test that provides an estimate of anthelmintic efficacy by comparing worm egg counts from animals before and after treatment [13]. The McMaster technique is widely used in veterinary parasitology and has been recommended by the WHO for evaluation of the EPG count in humans [29]. The FLOTAC technique was used in only one study [30]. FLOTAC and Mini-FLOTAC techniques [31, 32] present potential for the qualitative and quantitative copromicroscopic diagnosis of parasites in a practical and simple way, and should be considered.

In 17.5% of the studies (7/40), the faecal culture technique was not carried out to identify the nematode genus that had infected the animals. This is a matter of great importance in assessing the BZ resistance in animal nematodes, because the diagnosis based on egg observation does not indicate the parasite genus involved, and in mixed infections, only one species may be resistant to the BZ drug [33]. In addition, only one study conducted an in vitro test – the egg hatch test - to detect the nematode species involved in BZ resistance. The egg hatch test can be used for detection of BZ resistance by assessing the drug’s ability to inhibit embryonation of the parasite [13, 34].

The molecular signature of BZ resistance in nematodes is the presence of SNPs in the β-tubulin isotype 1 gene in nematodes, located at codons F167Y (TTC/Phe → TAC/Tyr), E198A (GAG/Glu → GCG/Ala) and F200Y (TTC/Phe → TAC/Tyr) [6, 35, 36]. Despite the fact that these genetic markers of BZ resistance are known, few studies (4/43, 9.3%) used molecular techniques for the evaluation of resistance of nematodes in Brazil. Of these studies, all *Haemonchus* nematodes demonstrated the mutation F200Y. Additionally, the codon F167Y was found in *Haemonchus* parasites in two studies. These findings in Brazil are in agreement with previous studies, which demonstrated that the F200Y mutation is the most frequently found mutation associated with BZ resistance in nematodes, and has been described in various nematode parasites: *Haemonchus* [36], *Ostertagia* [37], *Cooperia* [38], *Ancylostoma caninum* [39], human hookworms and *T. trichiura* [10, 40]. Moreover, *Haemonchus contortus* has been determined to be responsible for the rapid development of BZ resistance in nematodes of small ruminants, probably due to its high genetic diversity and consequent greater availability for new mutations [41, 42].

## Conclusions

The scenario of BZ resistance in nematode populations of domestic animals in Brazil is not favourable. Given the high cost of drug discovery and development, it is urgent to implement control measures and to monitor the effectiveness/resistance to nematodes in livestock in Brazil. Considering the BZ-R scenario observed in this study, a greater investment in animal management and adequate control of the use of anthelmintic drugs should be performed in the country.

## Abbreviations

AL: Alagoas
ALB: albendazole
BA: Bahia
BZ: benzimidazole
CE: Ceará
epg: Eggs per gram of faeces
FECRT: Faecal Egg Count Reduction Test
FEN: fenbendazole
MEB: mebendazol
MG: Minas Gerais
MS: Mato Grosso do Sul
OXF: oxfendazole
PB: Paraíba
PE: Pernambuco
PR: Paraná
RIC: ricobendazole
RJ: Rio de Janeiro
RN: Rio Grande do Norte
RS: Rio Grande do Sul
SC: Santa Catarina
SNPs: Single nucleotide polymorphisms
SP: São Paulo
THI: Thiabendazole
